# Americans misperceive the frequency and format of political debate

**DOI:** 10.1038/s41598-024-55131-4

**Published:** 2024-03-06

**Authors:** Erica R. Bailey, Michael W. White, Sheena S. Iyengar, Modupe Akinola

**Affiliations:** 1 UC Berkeley, Haas School of Business, Berkeley, USA; 2 Columbia University, Columbia Business School, New York, USA

**Keywords:** Psychology, Human behaviour

## Abstract

Disagreement over divergent viewpoints seems like an ever-present feature of American life—but how common is debate and with whom do debates most often occur? In the present research, we theorize that the landscape of debate is distorted by social media and the salience of negativity present in high-profile spats. To understand the true landscape of debate, we conducted three studies (*N* = 2985) across online and lab samples. In contrast to the high-profile nature of negative debates with strangers, we found that people most commonly debate close contacts, namely family members and good friends. In addition, they often report feeling positive after engaging in debate. We then directly measured misperceptions regarding debate in a representative sample of Americans (*N* = 1991). We found that Americans systematically overestimated how often others engage in debate. This overestimation extended across debate partners (family members, good friends, acquaintances, coworkers, and strangers) and contexts (in-person and online; *p’*s < 0.001, *d’*s > 0.98), most strongly overestimating how often Americans debate strangers online. This misprediction may be psychologically costly: overestimating how often Americans debate strangers online significantly predicted greater hopelessness in the future of America. Together, our findings suggest that Americans may experience a false reality about the landscape of debate which can unnecessarily undermine their hope about the future.

## Introduction

At first blush, the American political landscape can seem quite bleak, in part because of heightened political polarization^1–3^. One way to remedy polarization is the use of productive political debate to close the gap between dissenting parties. Rather than focusing on interventions to make debates more productive, the present research takes a step back, asking is the experience of debate for ordinary citizens well-understood? We suggest that three forces—the salience of online debates, the amplification of negative content online, and a negativity bias in human information processing—have together warped perceptions of how debate actually occurs among everyday Americans.

In this article, we begin to explore the landscape of debate using self-reported experiences regarding the topics, format, and partners of debate. In addition, we explore the warped perception of the same. To do this, we examine freely-recalled debates as well as personal experiences with debates, including topics, format, feelings, and frequency. We then directly examine misperceptions of the debate landscape. Finally, we tie these misperceptions to an important outcome: the overestimation of online debates with strangers is correlated with increased hopelessness for the future of America. These findings reposition empirical research towards more phenomenologically aligned settings and experiences with important implications for how to make debates better. The first step in improving productive political debates is understanding how, where, and with whom debates occur.

### Background

Productive debate has been suggested as an essential step towards solving the growing issue of political polarization^4,5^ which promotes and exacerbates disagreement around social and economic issues. To make progress on these issues, people from opposing sides often are tasked with coming to some form of agreement regarding a path forward. A common method for achieving consensus is to engage in a debate, where the ideas and positions of each party are discussed to arrive at a common endpoint. In academic research, debate is typically considered a communication process through which opinions on public affairs can be formed^6^. To better understand how individuals construe a debate, we asked a sample of online participants to define debate. Synthesizing these responses, we define a debate as a "discussion about a specific topic or issue that involves at least two different points of view" (see Supplemental Study 1 for more details). Debates can range from highly structured, such as Presidential election debates, to completely unstructured and self-guided such as debates occurring on social media platforms like X, TikTok, and Facebook. In addition, these debates can be very public and personal and range from heated dinner discussions with distant relatives to anonymous arguments in online forums.

To better quantify and understand the landscape of debate, social scientists have turned to social media platforms as a rich source of data. Researchers have examined debates over hot-button issues in the U.S. and abroad, including COVID-19^7^, Brexit^8,9^ and the January 6th insurrection^10^. The website X (formerly Twitter), in particular, is often the focus of this research. The functionality of X, in particular the use of hashtags, allows for “instantaneous debate and commentary about virtually every subject under the sun”^11–13^. Additionally, researchers seeking to analyze debates are aided by the ease of accessing data through APIs^14^.

However, conclusions drawn from online data sources are limited in generalizability given the non-representative nature of social media users^15–17^. In addition, within those who are on these sites, the use of the platform is also unequal, with a few users producing most of the content, particularly when that content is highly political^18^. Moreover, to the extent people engage with social media, this vehicle is merely one of many in the average American’s media landscape^19^. Therefore, the average American likely encounters debate topics online, as well as through myriad other sources. As such, we reason that debate may be misperceived, particularly the prevalence of online debate, as a result of the way information flows online.

There is evidence that negative information spreads more quickly on social media and is often amplified by social media algorithms that promote or push content to the forefront of users’ pages^20–22^. This negativity is exacerbated by non-human actors or “bots” that often inflame online conflicts^23^. When people do perceive information on social media, they also tend to view it more negatively as a result of being observed on the platform^24^. These factors combined suggest that negative, conflict-laden debates will flow to the top of people’s timelines. Even worse, when this information is observed, psychological research suggests that this negativity will make information more “sticky,” both highly salient and more accessible in people’s minds^25–27^. This line of reasoning is consistent with theorizing and empirical work on deliberative democracy which, although emphasizing the crucial place of deliberation and discussion in democracy, also recognizes that these experiences of discussion, dialogues, and debate amongst citizens are often negative, challenging, and rife with conflict^28–30^. Taken together, platform effects boosting negative content as well as the psychological processes that influence how this information is processed suggest that debate may be overestimated in its frequency.

Importantly, misperceptions of the frequency of debate may be psychologically costly. We argue that when individuals overestimate how frequently debate occurs, debate can appear costly and ineffective, decreasing hope for America’s future. Relatedly, being critical of a functioning political system has been shown to predict political hopelessness^31^. If this is the case, being hopeless about the future of America could have important implications for democratic expression. Past research has found that hope in the political process predicted self-reported voting behavior in the 2020 election^32^. In addition, hope is crucial in predicting whether people engage in collective action^33^.

## Results

To begin to understand the landscape of debate, Study 1 asked an online sample to freely recall a recent debate (*N* = 282). Participants then evaluated the characteristics of that debate. We were interested in whether participants reported these debates being generally negative (or positive) and whether they believed the debates to be representative of the average debate. We found that around half of these debates (47.87%) were observed online (vs. in person), with participants characterizing these debates as more negative than positive (*p* < 0.001, Cohen’s *d* = 0.47). In addition, participants believed these debates were representative of the average debate (*p* < 0.001, Cohen’s *d* = 0.52). Taken together, freely recalled debates are often observed online and seen as negative, characteristics that participants believe are representative of the average debate.

**Figure 1:**
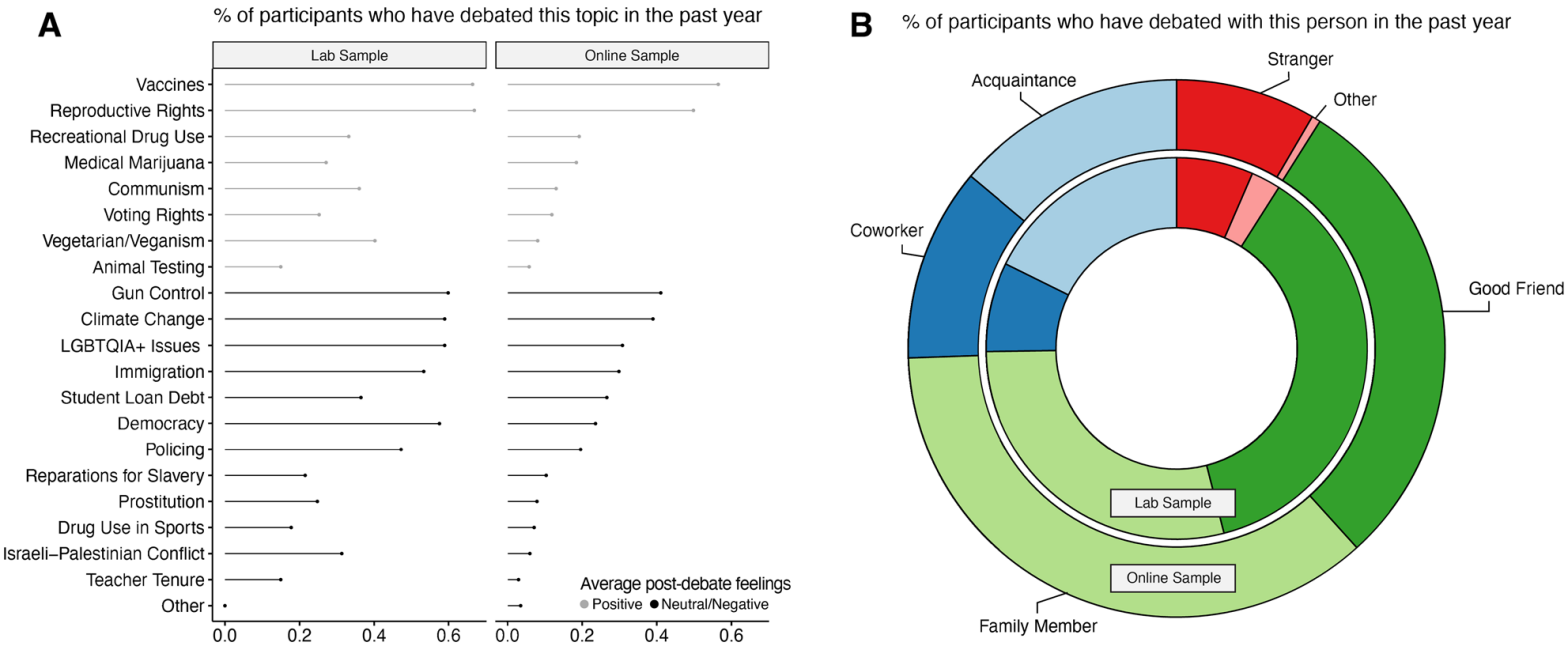
Topics and debate partners in lab and online samples. Figure presents the results of Studies 2a-2b which asked participants about their experience debating a series of issues over the past year. Panel (**A**) displays the frequency of debate topics in our sample with the most common debate topic being vaccines. Gray lines indicate debates that the average respondent felt positive after this debate; black lines indicate that the average respondent felt neutral-negative following this debate. Panel (**B**) displays debate partners with the most common partner in both samples being family members followed by good friends.

To link these perceptions with the landscape of debates, we turn to people’s self-reported experience with debates. This approach begins to answer a call for those interested in deliberative democracy to observe what debate looks like for ordinary individuals^34^. Studies 2a-2b surveyed two samples, asking them to self-report their experience with debate including questions regarding the topics debated, their relationship to the person with whom they debated, and their post-debate feelings (see Fig. 1). Study 2a consisted of 215 participants recruited from a behavioral science research laboratory. Study 2b consisted of 526 participants recruited from Amazon’s Mechanical Turk. In both samples, participants were presented with a list of 20 common debate topics and asked to select the topics they have debated about in the past year. For each debate topic they selected, they were asked to indicate the groups of people with whom they had this debate. Finally, they were asked to indicate how they felt following the debate.

The most common debate categories in our samples were “reproductive rights” and “vaccines” with 66–67% of the sample debating one of these issues in the past year, aligned with major news events around COVID-19 boosters and overturning of Roe v. Wade. Other high-profile debate topics were less common, such as policing (lab: 47%, online: 20%), and immigration (lab: 53%, online: 30%).

Overall, the average percentage of debates across topics was below 50% (lab: 37%, online: 20%) indicating that the majority of high profile debate topics are debated by less than half o participants in a 12-month period. The lab sample, consisting of a majority of university students, reported debating significantly more often across topics compared to the online sample (*p* = 0.005; Cohen’s d = 0.95).

In contrast to the high-profile nature of Twitter debates, we found that the most common debate partners tend to be close contacts, namely family members, and good friends. In both samples, of the topics people debated, these two categories accounted for more than half of the overall debates that participants engaged in (lab: 66%, online: 66%), while debates with strangers were relatively infrequent (lab: 7%, online: 8%).

Interestingly, when we examined participants’ post-debate feelings, we found that not all debates left participants feeling negative. In fact, in the online sample, the average feeling following a debate across topics was positive, being significantly higher than the midpoint of the scale (*p* < 0.001; Cohen’s d = 0.90). In the lab sample, post-debate feeling was not significantly different than the midpoint of the scale, indicating it was neither positive nor negative (*p* = 0.148). There was heterogeneity in feelings across debate topics with some debate leaving participants feeling more positive (voting rights, medical marijuana policies, animal rights) than others (policing, reparations, immigration).

Taken together, these findings suggest that the “typical” debate seems substantively different than two strangers typing at one another from behind their computer screens. Thus, the goal of Study 3 was to quantify the extent to which this misperception exists in the minds of everyday Americans and to examine if there are correlated consequences of holding these misperceptions.

Study 3 was a preregistered, nationally representative sample of Americans (*N* = 1989) recruited from Prolific Academic. Participants were randomly assigned to one of two conditions, either completing the survey as an *experiencer* or a *predictor*. Experiencers in our sample self-reported their experiences with debate in two formats—online and in-person. They also reported with whom they had these debates. Predictors were asked to accurately predict the percentage of respondents who had specific debate experiences. We were then able to compare the percentage of respondents who *experienced* specific types of debates with the *perception* of those rates. In addition, we examined an important outcome of this misperception: hopelessness. We hypothesized that the extent of the misperception would be tied to greater hopelessness in the future of America.

Across debate partners and formats, we found a significant overestimation regarding the frequency of debates. Perceivers overestimated the frequency of debates in both formats across all debate partners with one exception (in-person debates with family members, *p* = 0.071; see Table 1). These results suggest that there is widespread misperception about the frequency of debates the average American is having.

**Table 1:** Actual, predicted and misperceptions of debate partners and formats.

	In-person debates				Online debates			
	Actual	Predicted	Misperception		Actual	Predicted	Misperception	
	%	%	t(992)	d	%	%	t(992)	d
Family member	53.11	54.60	1.81	0.06	12.65	37.12	30.82***	0.98
Good friend	32.43	46.24	17.09***	0.54	19.58	37.40	22.08***	0.70
Acquaintance	8.33	34.15	33.68***	1.07	9.34	38.75	37.31***	1.18
Coworker	13.96	39.90	34.08***	1.08	4.42	24.91	31.01***	0.98
Stranger	4.02	19.21	25.47***	0.81	20.18	46.83	31.11***	0.99

Table displays the actual percentage of participants who experienced a debate with the target person in an in-person format (left) or online format (right). The *t* statistic and Cohen’s d effect size is based on a one-sample *t* test comparing the predicted percentage to the actual percentage. ***p < 0.001.

**Figure 2:**
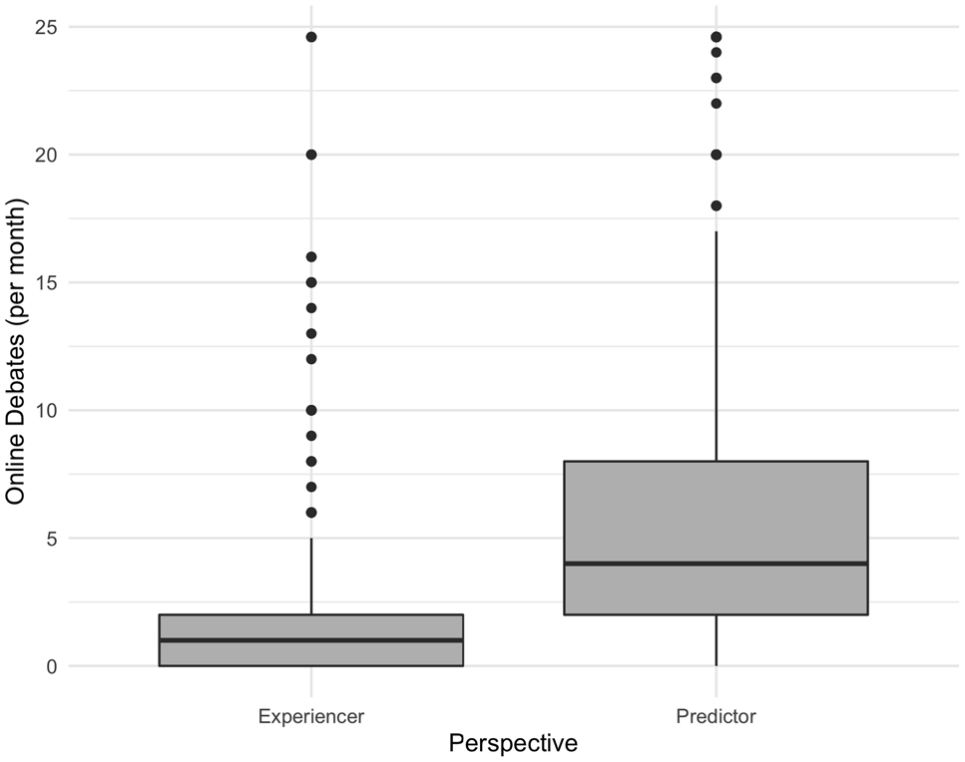
Overestimation of monthly debates. Figure displays responses to the open text box question regarding how many debates participants had engaged in in the past month. On the left is the self-reported experience of participants; on the right is the predicted number of debates for the average person. The plot consists of a rectangular box that spans the interquartile range (IQR) of the data, with a vertical line inside the box representing the median. The whiskers extend from the box to the minimum and maximum values of the data, excluding any outliers, which are plotted as individual points.

In addition, participants responded to an open text box where they were asked to report the number of debates they had engaged in in the past month (experiencers) or predict how many debates the average respondent had engaged in (predictors). We found that there were outliers in our dataset, so in a deviation from our preregistration, we winsorized the numeric responses to this question. We then compared the difference between the two conditions using a t-test. We found that there was a significant difference such that predictors expected a higher frequency of debate (M = 6.89) relative to the self-reported experience of participants (M = 1.50; mean difference = 5.39, 95% CI [4.91, 5.87], t(1313) = 22.14, *p* < 0.001; Cohen’s d = 0.99, 95% CI [− 1.09, − 0.90]; see Fig. 2).

Finally, we examined a negative outcome associated with this misperception. Specifically, we were interested in how hopeless participants felt about the future of America. We found that the perceptions regarding how often people debate strangers online significantly predicted hopelessness regarding the future of America (*Std*. *beta* = 0.17, 95% CI [0.11, 0.23], *t*(991) = 5.35, *p* < 0.001).

## Discussion

To better understand the landscape of debate in American life, we surveyed four independent samples about their beliefs, experiences, and predictions about debate. In Study 1, we found that the most salient debates as described by our participants were negative in nature and about half of these debates were observed in an online context. In addition, participants believed that these debates were representative of the “typical” debate. In Studies 2a-b, we focused on observing the experience of debates in both a lab and online sample. In both samples, we found that the most common debate experience was with close friends and family members. Study 3 measured the precise nature of the misprediction by randomly assigning participants to report their experience or their prediction about others’ experience of debates. In this sample we were able to directly observe the misprediction, finding that people overestimated the frequency of debates in almost every category of debate partner and in both online and in-person formats.

Our research has several contributions. First, we provide clarity on the landscape of debate experiences for Americans. This finding is in line with recent theoretical frameworks that highlight how and when people engage in political debate—specifically finding that people find political debates to be unpleasant, challenging, and anxiety-inducing experiences^35^. Indeed, documenting the relatively rare occurrence of debates emphasizes a broad consequence of the attention social media debates receive: Americans’ expectations of how often people debate were generally miscalibrated to reality—an effect that was particularly consequential in the context of online debates. Our findings suggest that the undue attention to online debates may exacerbate the average American’s feelings of hopelessness in the future of the country. Finally, our findings have implications for social science research. When empirical research tends to focus on online contexts for debate specifically around political issues, findings may be skewed. They may also fail to yield productive value if the majority of debates are had in person and with those of like-minded political affiliations^35^.

This research has a number of limitations that offer possibilities for future research. First, our source of “truth” regarding debate experiences come from self-reported experiences from participants. That is, we do not observe our participants engaging in these debates, rather rely on them to tell us what debates they have. One way to avoid this issue would be to conduct a longitudinal study where debates can be directly observed. Here, we elected to compare self-reported experiences to perceptions of the same as a way of precisely measuring the misperception. By comparing responses to the same items, randomly assigned at the recruitment level, we can minimize issues with regards to selection into a longitudinal study relative to an online survey (for example). However, we encourage future researchers to measure debates in a more ecologically valid way. Further, for our experiencers, negative debates may be more memorable or seem more frequent. If this is the case, our effects may be conservative estimates of true debates as the negativity makes them seem more ever-present. This issue is partially addressed by the self-reported positivity of debates (lab sample) and neutrality of debates (online sample), however without perfect observation of these debates, the representative nature of the responses is unclear. Relatedly, an additional limitation of our findings is that we recruited participants from a lab sample on an American university campus and a representative sample from Prolific Academic. Both samples are non-representative of the full population of Americans. We encourage future researchers to replicate or extend these findings using a survey firm that could extend the target and breadth of the empirical findings.

An additional limitation is that our findings are largely correlational, in particular the relationship between misperceiving debates and hopelessness. Thus, it is unclear whether addressing this misperception could reduce feelings of hopelessness. Related research has found that addressing these issues is not necessarily as simple as correcting the misperceptions^36^. Thus, rather than correcting the misperception itself, future research could instead manipulate whether participants observe a particularly negative or positive debate and measure the impact on hopelessness.

Finally, while our findings speak to the general landscape of debate, they do not provide insight into why Americans choose to debate different partners. There may be an interaction between debate topic and format which predicts debate partners; this choice may therefore predict post-debate feelings. Further, we examine post-debate feelings but not post-debate learnings. Future research should examine whether feeling positive following a debate correlates with whether participants increased their factual understanding of an issue, their feelings towards the political outgroup, and willingness to approach future political debates.

Future research should also consider the ways that political debate ebbs and flows in everyday life. For instance, it is likely that debate perceptions and experiences change over time. For instance, both perceptions and experiences may fluctuate in line with election cycles^37^, as candidates in political campaigns bring issues to the forefront of the public discourse. Further, we do not consider the important outcomes associated with engaging in debates, however frequent. Considering that past research has found that engaging in debates is positively correlated with a better understanding of both sides of an issue^38^, future work may consider ways to increase experiences of debate outside of an online context.

Debate is ostensibly common amongst Americans; however, we find that this perception is significantly higher than reality—and this overestimation may be psychologically costly, coming at the expense of hope about the future of America.

## Methods

### Study 1

#### Preregistration

Data collection, measurement, and analysis follow our preregistration plan which can be found here: https://aspredicted.org/b9hm6.pdf.

#### Participants

Three hundred participants were recruited for this study from Amazon’s Mechanical Turk. In total, we received 297 responses. After removing participants who did not pass our data quality checks as preregistered (*n* = 15), our final sample consisted of 282 participants (144 men, 137 women, 1 non-binary individual; average age = 40.74 years, SD = 11.70 years; 78.72% White, 8.87% Black or African American, 6.03% Asian, 4.96% Hispanic or Latino/a, 2.84% Other).

#### Procedure

We asked participants to “Think about a debate you have seen or witnessed recently. What was the topic of that debate?” They answered in an open text box. They were then asked to describe who participated in the debate and where they observed the debate. They answered these questions using two open text boxes.

#### Measures

We were interested in two key dependent variables regarding this recalled debate. First, we were interested in how negative (vs. positive) the debate was. We had hypothesized that because negative events are easier to recall, participants would be more likely to recall negative debates. We measured this by asking participants, “How would you characterize the tone of the debate?” They responded on a 7-point scale where 1 = *Extremely negative* and 7 = *Extremely positive* (M = 3.31, SD = 1.47).

Second, we were interested in how representative they believed this debate to be. To measure this, we asked participants, “Would you say this debate is reflective of the typical or average debate?” They responded on a 7-point scale where 1 = *Not at all reflective* and 7 = *Very reflective* (M = 4.76, SD = 1.46).

#### Additional results

First, we examined the topics of the debates that participants recalled. We found that the most debated topic was gun control (18.09%), followed by political issues including local races (e.g. Chicago mayoral race) and country-wide issues (e.g., January 6th insurrection; total 16.31%), and reproductive rights (9.57%).

We then tested our key dependent variables. We theorized that debates that were freely recalled by participants would be more negative in nature relative to positive. To test this, we conducted a one-sample *t*-test comparing participant responses to the midpoint of the scale (4). We found that participants characterized these debates as significantly more negative than the midpoint of the scale (difference = − 0.69, 95% CI [3.14, 3.48], *t*(281) = -7.91, *p* < 0.001; Cohen’s d = − 0.47, 95% CI [0.59, 0.35]). Our second key dependent variable was how representative participants believed this debate to be. To test this, we compared the average response to the midpoint of the scale (4). We found that participants viewed the debate as representative of the average debate (difference = 0.76, 95% CI [4.59, 4.93], t(281) = 8.77, *p* < 0.001; Cohen’s d = 0.52, 95% CI [0.40, 0.65]).

### Studies 2a-2b

#### Study 2a participants

Two hundred and fourteen participants were recruited for this study from a research lab at a large Northeastern university. We did not collect demographic information from participants directly, however demographic information was provided for a subset of participants from the research lab (67 men, 127 women, 1 non-binary individual, 1 other-identifying individual; average age = 23.34 years, SD = 5.29 years; 56.63% Asian, 27.04% White, 10.20% Hispanic or Latino/a, 6.12% Black or African American, 8.16% Other; Participants were able to select one or more race/ethnicities).

#### Study 2b participants

Five hundred participants were recruited for this study from Amazon’s Mechanical Turk. We did not collect demographic information from participants directly, however, demographic information was provided for a subset of participants from MTurk (145 men, 138 women, 217 did not report).

#### Procedure

To compare the frequency of debate topics in a systematic format, we presented participants with a list of twenty common debate topics. From this list, they were asked to indicate whether they have had a debate on that topic in the last year. The list of debate topics included: Abortion and/or Reproductive Rights, Animal Testing, Climate Change, Communism, Democracy, Drug Use in Sports, Gun Control, Immigration, Israeli-Palestinian Conflict, Medical Marijuana, Policing, Prostitution, Recreational Drug Use, Reparations for Slavery, Sexual Orientation and/or Gender Identity Issues, Student Loan Debt, Teacher Tenure, Vaccines, Vegetarian/Veganism, and Voting Rights. In addition, participants were given the option of “Other” with an open text box to capture debate topics not featured in our list.

#### Measures

For each debate topic participants indicated they had debated, they were asked, “You mentioned the topic of [Topic], with whom did you have the debate about [Topic]?” They then selected as many of the following as applied: A family member, A good friend, An acquaintance, A coworker, A stranger, or Someone else. To capture participant’s feelings following the debate, they were asked, “Following the debate about [Topic] how did you feel?” They responded on a 7-point scale where 1 = *Extremely bad* and 7 = *Extremely good*.

### Study 3

#### Preregistration

Data collection, measurement, and analysis follow our preregistration plan which can be found here: https://aspredicted.org/rs8nj.pdf.

#### Participants

Two thousand participants were recruited for this study from Amazon’s Mechanical Turk. As preregistered, we kept the survey open for two weeks. At the end of 2 weeks, we received 1989 responses. All participants passed an attention check (957 men, 999 women, 23 non-binary individuals, 10 other-identifying individuals; average age = 45.15 years, SD = 15.95 years; 76.07% White, 13.32% Black or African American, 5.48% Asian, 4.07% Hispanic or Latino/a, 1.26% Other).

#### Procedure

Participants were randomly assigned to one of two conditions, an *experiencer*, or a *predictor*. These conditions filtered participants into one of two arms of the survey with a different set of questions for each. *Experiencers* reported their own experience of debates. *Predictors* were tasked with estimating the percentage of *experiencers* that would report a set of specific debate experiences. Thus, we were able to directly compare the experience of debate to the prediction of the same.

#### Measures

##### Experiencer-debate partners

We asked experiencers to indicate the person(s) they had debated in the last month. They were asked, “In the last month, with whom of the following have you had a debate with [in-person/online]? Please select all that apply.” The options were: Family member, Good friend, Acquaintance, Coworker, Stranger, and None.

##### Experiencer-debate frequency

*Experiencers* reported the total number of online and in-person debates they had engaged in in the last month. The items read, “How many [online/in-person] debates have you had in the last month?”.

##### Predictor survey instructions

To ensure that participants in the *predictor* condition understood the format of their questions, we provided them with the following instructions: “We previously surveyed a representative sample of 1000 Americans from Prolific about their personal experience with debates. Your goal in this survey is to accurately predict what percent (%) of Americans had each debate experience from 0 to 100%. For example, if we asked you, “What percentage of participants reported that they had engaged in a debate about whether pineapple belongs on pizza?”: Responding with 0% would indicate that you believe 0 of the 100 Americans surveyed had debated whether pineapple belongs on pizza; Responding with 50% would indicate that you believe 50 of the 100 Americans surveyed debated whether pineapple belongs on pizza; Responding with 100% would indicate that you believe 100 of the 100 of surveyed Americans debated whether pineapple belongs on pizza”.

In addition, to incentivize accuracy in their responses, we told participants that the participant with the most accurate predictions across each debate experience would win a $100 bonus.

##### Predictor-debate partners

For each debate partner category, participants assigned to the *Predictor* condition predicted what percent of Americans had engaged in each of the types of debate within the last month (0 to 100%).

##### Predictor-debate frequency

*Predictors* were asked to predict the total number of online and in-person debates the average American had in the last month using the following items, “How many [online/in-person] debates did the average American have last month?”.

##### Hopelessness

We also collected a measure of hopelessness as a potential consequence of debate frequency misperception. We measured hopelessness, specifically regarding the future of America, by adapting a four-item measure of hopelessness from past research^39^: “I have great faith in the future of America,”* “When I look ahead to the future, I expect America will be better than it is now,”* “The future of America seems vague and uncertain to me,” and “America’s future seems dark to me” Participants responded on a 7-point scale where 1 = *Strongly disagree* and 7 = *Strongly agree* (* = reverse-coded, *α* = 0.90).

### Ethics statement and reproducibility

All studies were approved by Columbia University’s institutional review board. All methods were performed in accordance with the relevant guidelines and regulations. All participants provided informed consent and were compensated for their participation. Materials, anonymized data (including descriptions of how the original files were anonymized) and analysis code for our research can be found at: https://osf.io/zkxtq/. The preregistration for Study 1 is available at: https://aspredicted.org/b9hm6.pdf; The preregistration for Study 3 is available at: https://aspredicted.org/rs8nj.pdf; Studies 2a-2b were not preregistered.

### Supplementary Information

Supplementary Information.

## Data Availability

The datasets generated and analyzed during the current study are available in the paper’s OSF repository: https://osf.io/zkxtq/?view_only=f5a8de80744d4c299d346f54c5a4e551.
