# Supplementary material for: Americans misperceive the frequency and format of political debate
Source: Sci Rep. 2024 Mar 6;14:5565. doi: 10.1038/s41598-024-55131-4 (PMC10918165; doi:10.1038/s41598-024-55131-4)
Supplement: Supplementary file 1 — Supplementary Information. [file 41598_2024_55131_MOESM1_ESM.docx]

#

**Supplemental Material for:**

**Americans Misperceive the Frequency and Format of Political Debate**

# Supplemental Study 1: What is a Debate?

The goal of the first section of this paper was to understand what people precisely mean when they talk about debate. In particular, we were interested in a lay definition of debate, where ordinary Americans are surveyed regarding how they would define a debate. We then conducted linguistic analysis, specifically topic modeling, to understand the key components of the definition. This allows us to synthesize the meaning of debate to use in future theoretical and empirical studying.

## Methods

Participants**.** We recruited 250 total participants from Amazon’s Mechanical Turk. In total, we received 249 total responses. All participants passed an attention check (“Which of the following is not a color?” Answers: Orange, blue, red, and bike). Therefore, our final sample consisted of 249 participants (129 men, 116 women, 1 non-binary individual, 2 other-identifying individuals; average age = 39.66 years, *SD* = 11.19 years; 79.52% White, 8.84% Black, 4.82% Latino/a, 4.42% Asian, 2.41% Other).

Procedure**.** Participants were recruited to this study to answer questions regarding debates. They were told, “In this short survey, you will tell us about your feelings and opinions towards debate.” The key question for this section was, “How would you define **debate**?” They answered this question using an open text box.

## Results

To analyze the data, we conducted topic modeling on the responses. Specifically, we used a “bag-of-words” approach which ignores the order of words used in each definition. We then created a hierarchical probabilistic model Dirichlet bigram language model of the *n-*grams, or stem of words. Our model hyperparameters were inferred using a Gibbs algorithm.

We used a number of metrics to analyze the optimal number of topics (Arun et al., 2010; Cao et al., 2009; Deveaud et al., 2014; Griffiths & Steyvers, 2004). This analysis revealed that four topics fit the document term matrix the best. Table 1 displays the most common *n*-grams in each topic.

**Table 1.** Topic Modeling Results, S*upplemental S*tudy 1

| **Topic 1** | **Topic 2** | **Topic 3** | **Topic 4** |
| --- | --- | --- | --- |
| “two” | “peopl” | “view” | “debat” |
| “tri” | “opinion” | “parti” | “oppos” |
| person” | “discuss” | “side” | “argument” |
| “topic” | “one” | “topic” | “two” |
| “think” | “side” | “viewpoint” | “subject” |
| “way” | “point” | “issu” | “formal” |
| “differ” | “idea” | “other” | “express” |
| “posit” | “inform” | “convinc” | “anoth” |

*Notes.* Table 1 features the top *n-*grams in each topic.

We synthesized the results of these topics into a definition of debate as follows: “a debate is a **discussion** about a **specific topic or issue** that involves **at least** **two** **different** **points of view.”** First, Topic 1 is captured in the fact that there are “at least two” points of view expressed in a debate. Two is the minimum number of parties which qualify a conversation into a debate because there must be “difference”. Thus, an individual can be in a debate with themselves to the extent there are at least two sides to the argument or issue at hand.

Relatedly, Topics 2 and 4 emphasize that these “sides” of the debate must express their point of view. They do this by debating, opposing, or arguing (Topic 4) their opinion, side, ideas or point (Topic 2). This is crucial as a debate does not occur simply when there are differing points of view, rather these points of view need to be expressed in some way. We take the neutral stance, using the word “discussion” in our definition to avoid biasing our respondents towards more negative forms of debate. Finally, Topic 3 notes that a debate is typically centered around a central issue, topic, or viewpoint. We reflect this in our definition with the phrase “specific topic or issue.”

**Supplemental Study 1 Discussion**

As a crucial preliminary step towards our understanding of the landscape of debate, we asked a set of online participants how they would define debate. We then conducted linguistic analyses, specifically topic modeling, to extract the key features of these definitions. From this analyses, we created our working definition of a debate.
